# Students Have Changed; Medical Teachers Should Change Too

**DOI:** 10.1055/s-0044-1793818

**Published:** 2024-11-20

**Authors:** Surajit Bhattacharya, Kaushik Bhattacharya, Neeta Bhattacharya

**Affiliations:** 1 Division of Plastic Surgery, Department of Plastic Reconstructive and Aesthetic Surgery, Ajanta Hospital, Lucknow, Uttar Pradesh, India; 2 Department of Surgery, Mata Gujri Memorial Medical College and Lions Seva Kendra Hospital, Kishanganj, Bihar, India; 3 Department of Radiodiagnosis, Medanta Hospital, Lucknow, Uttar Pradesh, India

*Today's students have not just changed incrementally from those of the past, nor simply changed their slang, clothes, body adornments, or styles, as has happened between generations previously. A really big discontinuity has taken place. One might even call it a “singularity” – an event which changes things so fundamentally that there is absolutely no going back. This so-called “singularity” is the arrival and rapid dissemination of digital technology in the last decades of the 20th century* .

–Marc Prensky

Apart from being an academic teacher par excellence, a medical college faculty member must take on multifaceted roles including scheduling the annual timetable and serving as syllabus coordinator, clinician, undergraduate/postgraduate internal and external examiner, mentor, facilitator, researcher with good publication, postgraduate dissertation guide, and conference delegate/speaker. 1 Each new generation of students will bring changes to teaching and learning. Medical teachers continually find themselves adapting to the instructional style to account for their interests, struggles, and goals. But one thing that will not change is the trend toward a more digital world. As the educational landscape continues to evolve, teachers are faced with the task of understanding and adapting to the unique characteristics of the Generation Z (Gen Z) students they teach today. Gen Z students possess distinct traits shaped by their experiences with technology, globalization, artificial intelligence, and social media. As teachers, understanding these traits is crucial for fostering an inclusive, engaging, and effective learning environment.

## Gen Z Students Are Digital Natives

Google, Instagram, Twitter, Facebook, YouTube, WhatsApp, and smartphones are not just convenient tools—they are necessary parts of life through which medical students stay connected to the world and are able to access information at any time. Gen Z students want immediate feedback on assignments just as they do on social media. They also crave autonomy in their education. Students want to make decisions about what they learn, when they learn, and how they learn and demonstrate their knowledge. The effective use of social media in medical education was felt by teachers during the Covid-19 pandemic. 2 The educational goals of using technology in medical education include facilitating basic knowledge acquisition, improving decision-making, enhancement of perceptual variation, improving skill coordination, practicing for rare or critical events, learning team training, and improving psychomotor skills. 3 These new generation of learners are called as “digital natives,” a phrase coined by Prensky. 4

### How Can Teachers Adapt?

Using educational software and medical apps, beginning a dialog online, using audiovisual media to advantage, and providing rationale and value for time are the challenges that medical educators face today.

Rather than trying to draw students away from technology, consider how one can use it to provide information and engage with them. Use of educational software and medical apps make the teacher's job easier and keep Gen Z students engaged. In addition to full-service learning management systems, the teacher can leverage software to create everything from interactive presentations to educational games. Apps like VOKA Anatomy Pro, Human Anatomy Atlas 2024, TeachMeAnatomy, Anatomy Learning - 3D Anatomy, and Biodigital Anatomy have made teaching anatomy both interesting and engaging. Long lectures are not the best technique for Gen Z students.

The Gen Z students are used to multitasking and skimming for the most valuable information. The teacher should encourage note-taking and engagement with classroom apps like Canvas. Lectures and large blocks of text can result in decreased student engagement. Using charts, graphics, and multimedia can make the material more memorable. For example, a whole chapter on velopharyngeal incompetence (VPI) is boring as compared with two nasoendoscopy and lateral fluoroscopy videos, one of a normal velopharynx and one with VPI and brief commentary.

Medical students prefer PowerPoint slides with clear organization, large, limited word texts, and visual aids such as images and animations. 5 It will be better to engage in online case discussions, seminars and journal clubs, and learning online from overseas experts. 6 YouTube videos, previously scrutinized by teachers, like Clinics@GK Hand Surgery, can be excellent learning tools ( https://www.youtube.com/c/GKHandSurgery ). 7

To encourage immediate feedback on the assignments as in the social media, Gen Z students can be offered a test comprising of 20 multiple choice questions (MCQs) on Google Form at the end of every class, so that they immediately get the feedback on how much they have understood the lesson. A recent study highlighted the efficacy of employing Google Form–based MCQ tests in enhancing students' comprehension of the biochemistry subject, evaluating their scores and improving the overall quality of learning as teachers were able to provide targeted feedback on areas that required improvement, thereby enhancing the learning experience. 8

Students want to make decisions about what they learn, from where they learn, how they learn, and how they demonstrate their knowledge. For students who are used to immediately reaching people online, sending an instant message is likely going to be more effective. Gen Z students are used to constantly updated newsfeeds and have come to expect only the most relevant information. Teachers should explain the rationale behind each query of the student. The materials on social media and the world wide web are usually unverified and unaccredited; therefore, students need rationale guidance of teachers to help them choose the correct bits.

## Gen Z Students Are Diverse

The student population is from diverse regions, cultures, and nationalities, and teachers have to be patient, inclusive, and always reassuring to counter mental health issues arising from academic pressure, high rates of perfectionism, financial worries, and insufficient sleep. Students compete in all-India competitive examinations to get selected and for many of them a new city with a new culture, new language, new food, and new set of rules can be overwhelming.

### How Can Teachers Adapt?

Interacting with individuals who are different helps people anticipate alternative viewpoints and recognize that reaching a consensus will take effort. That is clearly relevant to teachers. But educators need to take extra measures to support a diverse student population. Four tips from Imagine Learning are the following:

- By understanding the social interests, goals, and thought-patterns that influence our culture, the teacher can better identify personal biases and recognize the value of cultural background.
- Go beyond the surface and aim to understand how the diversity and cultural backgrounds of the students affect how they see themselves and the world around them.
- Language and dialect are key parts of a culture. By examining how words are used, the teacher can learn to communicate with students in a more meaningful way.
- Incorporating multicultural literature can help students identify more with the material and foster cross-cultural understanding.

## Gen Z Students Experience High Rates of Depression and Anxiety

It can be easy to dismiss a moody or withdrawn behavior as typical student angst. Potential contributing factors include academic pressure, high rates of perfectionism, anxiety of the social environment, burnout, and lack of adequate sleep. 9 All this is to say there is a high likelihood that the medical teacher will often encounter a student with a mental illness.

### How Can Teachers Adapt?

Teachers can do a lot to support students struggling with mental health issues. Rather than using an authoritative teaching style, the teacher should try engaging Gen Z students one on one and be sensitive that their emotions may impact their learning. This is not to say that the teacher should let bad behavior go unaddressed, but he or she can use strategies that focus on empowering students. Try rewarding engagement with verbal praise and regularly reviewing ways that students demonstrate growth. Educators can give assignment extensions, break tasks into smaller pieces, and offer to help students create study plans. They may also encourage students to help one another. Recognizing success can give students much-needed confidence. Even though one must follow state standards, the intelligent teacher can break up the curriculum into frequent milestones for students to celebrate. Mental health personnel can provide counseling, connect students to further services, and collaborate with family members. Finally, learning from experienced educators can help teachers to apply evidence-based research to their teaching.

Teachers should understand that they are not above personal biases themselves. They have to be appreciative of their own culture and at the same time try to understand the culture of the students coming from various parts of the country or even overseas. This itself builds trust and bond with students from distant places.

## Types of Competencies for Gen Z Students

As medical educators, we are trying to imbibe three types of competencies in our students: cognitive, interpersonal, and intrapersonal.

## Cognitive Competencies

These involve the following:

- Communicate effectively.
- Listen actively.
- Think critically.
- Reason logically.
- Interpret clearly.

## Interpersonal Competencies

Empathy is a cornerstone the 21st-century competency, as it allows students to consider the true complexity of issues. To achieve tolerance and respect for others, 21st-century learners need to master the following skills:

- Collaboration.
- Cooperation.
- Leadership.
- Responsibility.
- Assertive communication.
- Social influence.

## Intrapersonal Competencies

The ability to remain flexible and adaptable to change will be more important than ever for the next generation of medical professionals. Ethical consideration in medical practice is mandatory along with research orientation, especially to those who intend to become medical teachers in academic institutions in future. The motto should be to enhance the role from “publish or perish” to “publish and flourish.” The art of documentation and its relevance in patient management as well as understanding the ways to avoid medicolegal hassles and medical negligence cases should also be taught to the students. Finally, it is important to prepare the students for life after the institutional degree for private practice in remote areas.

To help students become comfortable taking initiative, persevering, and remaining innovative, 21st-century educators should work to nurture the following traits:

- Ethical orientation.
- Self-regulation.
- Intellectual openness.

## The 4Cs of Gen Z Medical Education

- ***Critical thinking and problem-solving:*** Arming students with strong critical thinking skills can help prepare them for success in higher education and the workforce alike. Becoming adept at critical thinking can be crucial in helping students develop other important skills like increased concentration, deepened analytical abilities, and improved thought processing. In our progressively automated society, the jobs that are least likely to become obsolete are the ones that require expert thinking and complex communication. Robust critical thinking abilities enable professionals to remain relevant for the longest period of time.
- ***Communication:*** To communicate clearly, medical students need to learn how to articulate their thoughts in a variety of contexts. They need to listen effectively and converse well in diverse environments using multiple media and technologies. Knowing how to validate their information sources, leverage information effectively, and use culturally effective communication will differentiate them from automated robots.
- ***Collaboration:*** Being able to work effectively with diverse teams, make compromises, and assume shared responsibility for work and take on the leadership mantle are essential for individuals to help their professional team meet their goals. The rise of technology and globalization in today's workforce has made these skills especially necessary. TEAM actually means Together Everybody Achieves More.
- ***Creativity:*** Innovation and creativity are also skills that can help professionals preserve their value amidst an increase in global competition and task automation. Our medical education should be such that students are inherently creative and have answers to every unexpected question or, at least, know where to search for them.

“That the great surgical teachers shine like stars in the rolling pitch-darkness of night, instead of limiting the challenges they come across; they by their dexterity, deftness and featliness challenge their limits and transcend all the formalistic barriers and enrich the posterity with innovative ideas and styles.” 10

## The Problem of New Age Learning

The new age methods mandate a fast internet connection, which might still be a luxury at many places. The digital divide can exacerbate existing inequalities, as students from low-income families may lack access to necessary technology and reliable internet connections. Online teaching could lead to isolation and disconnect from the teachers and the peers. Subjects that require practical, hands-on experience, such as laboratory work, art, or physical education, may be less effective in a virtual format. A blended learning model comprising face-to-face classroom teaching, combined with online resources, would allow students to review materials at their own pace and access additional resources.

## The Changing Role of a Teacher

The teacher is no more just a provider of knowledge. He or she today has a multifaceted role and is a communicator, a disciplinarian, a conveyor of information, an evaluator, a unit manager, a counselor, a member of many teams and groups, a decision-maker, a role model, and at times even a surrogate parent. Each of these roles requires practice and skills that are often not taught but are acquired by nurturing appropriate temperament, skills, and personality. Now when you add this burden of expectation to the changing attitude of the students, you truly start appreciating how much the student has changed and how urgently the teacher needs to change too.

## Conclusion

Gen Z medical students are digital natives, connected to the world by Google, Instagram, WhatsApp, and smartphones and able to access information at any time. To expect them to listen to hourlong didactic lectures is simply impractical. Their attention span is notoriously short, and their educational needs are better addressed by educational software, audiovisual media, and accredited online lectures and YouTube videos. So, rather than trying to draw them away from technology, educators have to consider how it can be used to provide information and engage with the students. All-India competitive entrance examinations have made the Gen Z student population very diverse and teachers will have to be patient, inclusive, and always reassuring to counter mental health issues rising from academic pressure, high rates of perfectionism, monitory worries, and lack of adequate sleep. Besides the subject being taught, teachers will have to teach skills like critical thinking and problem-solving, communication skills, collaboration skills, creativity, and innovation.
